# Tumor antigen glycosaminoglycan modification regulates antibody-drug conjugate delivery and cytotoxicity

**DOI:** 10.18632/oncotarget.16921

**Published:** 2017-04-07

**Authors:** Helena C. Christianson, Julien A. Menard, Vineesh Indira Chandran, Erika Bourseau-Guilmain, Dmitry Shevela, Jon Lidfeldt, Ann-Sofie Månsson, Silvia Pastorekova, Johannes Messinger, Mattias Belting

**Affiliations:** ^1^ Department of Clinical Sciences, Section of Oncology and Pathology, Lund University, Lund, Sweden; ^2^ CNRS UMR 5237 CRBM, Montpellier University, Montpellier, France; ^3^ Department of Chemistry, Chemical Biological Centre, Umeå University, Umeå, Sweden; ^4^ Biomedical Research Centre, Institute of Virology, Slovak Academy of Sciences, Bratislava, Slovakia; ^5^ Department of Chemistry – Ångström, Molecular Biomimetics, Uppsala University, Uppsala, Sweden; ^6^ Department of Oncology, Skåne University Hospital, Lund, Sweden

**Keywords:** tumor antigen, glycosylation, hypoxia, immunotherapy, proteoglycan

## Abstract

Aggressive cancers are characterized by hypoxia, which is a key driver of tumor development and treatment resistance. Proteins specifically expressed in the hypoxic tumor microenvironment thus represent interesting candidates for targeted drug delivery strategies. Carbonic anhydrase (CAIX) has been identified as an attractive treatment target as it is highly hypoxia specific and expressed at the cell-surface to promote cancer cell aggressiveness. Here, we find that cancer cell internalization of CAIX is negatively regulated by post-translational modification with chondroitin or heparan sulfate glycosaminoglycan chains. We show that perturbed glycosaminoglycan modification results in increased CAIX endocytosis. We hypothesized that perturbation of CAIX glycosaminoglycan conjugation may provide opportunities for enhanced drug delivery to hypoxic tumor cells. In support of this concept, pharmacological inhibition of glycosaminoglycan biosynthesis with xylosides significantly potentiated the internalization and cytotoxic activity of an antibody-drug conjugate (ADC) targeted at CAIX. Moreover, cells expressing glycosaminoglycan-deficient CAIX were significantly more sensitive to ADC treatment as compared with cells expressing wild-type CAIX. We find that inhibition of CAIX endocytosis is associated with an increased localization of glycosaminoglycan-conjugated CAIX in membrane lipid raft domains stabilized by caveolin-1 clusters. The association of CAIX with caveolin-1 was partially attenuated by acidosis, *i.e.* another important feature of malignant tumors. Accordingly, we found increased internalization of CAIX at acidic conditions. These findings provide first evidence that intracellular drug delivery at pathophysiological conditions of malignant tumors can be attenuated by tumor antigen glycosaminoglycan modification, which is of conceptual importance in the future development of targeted cancer treatments.

## INTRODUCTION

Aggressive tumors are characterized by poorly perfused, hypoxic and acidic niches that are resistant to conventional oncological treatment with cytostatic agents and radiotherapy. Recent advances in the understanding of how cancer cells adapt to hypoxic stress have the potential to significantly improve current treatment strategies. Hypoxia-induced carbonic anhydrase IX (CAIX) catalyzes the reversible hydration of carbon dioxide, and together with bicarbonate transport proteins facilitates intracellular alkalinisation with concomitant extracellular proton accumulation and acidification that significantly contribute to the invasive and metastatic potential of tumor cells. CAIX has thus emerged as an attractive treatment target due to its specific expression in the tumor microenvironment that further correlates with worse patient prognosis [1–6].

The concept of antibody-drug conjugate (ADC) treatment is to repurpose an antibody as a toxin delivery vehicle to specifically kill tumor cells by endocytic uptake and intracellular release of the drug. ADCs targeting epidermal growth factor receptor 2 (HER-2) and CD20 have been approved in the treatment of breast cancer and lymphoma, and more than 40 ADCs are currently tested in clinical trials [7, 8]. Tumor specific cell-surface proteins, such as CAIX, with endocytic transport activity are interesting targets for ADCs. Importantly, CAIX and other targets of therapeutic antibodies are plasma membrane resident proteins more or less modified by glycosylation, which is known to have a major impact on the sorting of cell-surface proteins [9], *e.g.* it was recently reported that tumor resistance to the EGFR-targeting antibody cetuximab correlates with reduced EGFR stability due to deficient glycosylation [10]. However, while considerable interest is focused on the optimal design of the targeting ADC, it remains unknown how tumor antigen glycosylation may dictate the efficiency of ADC based anti-cancer treatments.

Here, we provide novel insights into how CAIX is modified by glycosaminoglycan (GAG), and investigate at the mechanistic and functional level how this type of glycosylation may be involved in tumor antigen endocytosis with the aim to better understand how to target aggressive tumors.

## RESULTS

### Identification of CAIX as a hypoxia induced proteoglycan

**Figure 1:**
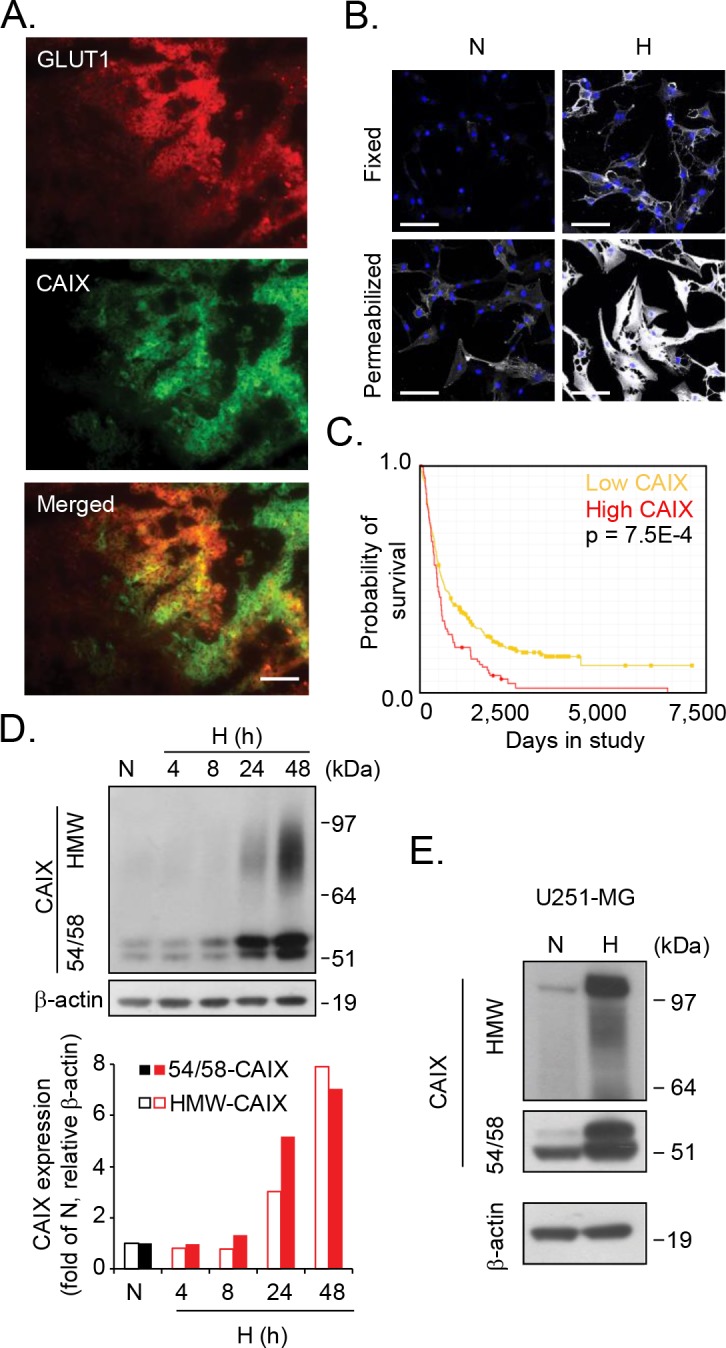
Identification of a heterogeneous, high molecular weight variant of CAIX **(A)**, Immunofluorescence staining of human glioblastoma tumor sections shows partial co-localization of GLUT1 (red) and CAIX (green) in merged images (yellow). Data shown are representative of three different tumors and at least five sections each. Scale bar, 50 μM. **(B)**, Confocal microscopy imaging of U87-MG glioblastoma cells grown under normoxic (N) or hypoxic (H) conditions for 72 h. Fixed (upper panel) and permeabilized cells (lower panel) were stained for CAIX (white) and Hoechst nuclear stain (blue). Scale bar, 50 μM. **(C)**, Kaplan-Meier survival plot for glioma patients with high CAIX gene expression (3≥-fold up-regulation, red line, n=50, compared with low CAIX, yellow line, n=342) demonstrates a correlation of high CAIX expression with worse patient outcome (p=0.00006). **(D)**, U87-MG cells were grown under normoxic (24 h) or hypoxic conditions for the indicated time periods. Upper panel: Immunoblotting for CAIX reveals a new CAIX variant. Lower panel: Quantitative data of CAIX/β-actin ratios of high molecular weight CAIX (HMW-CAIX) and 54/58 kDa CAIX (54/58-CAIX) at normoxia and various periods of hypoxia. **(E)**, U251-MG glioblastoma cells were grown under normoxic or hypoxic conditions for 48 h, and cell lysates were analyzed for CAIX and β-actin expression by immunoblotting. **(D, E)** Shown are representative immunoblots from three independent experiments.

**Figure 2:**
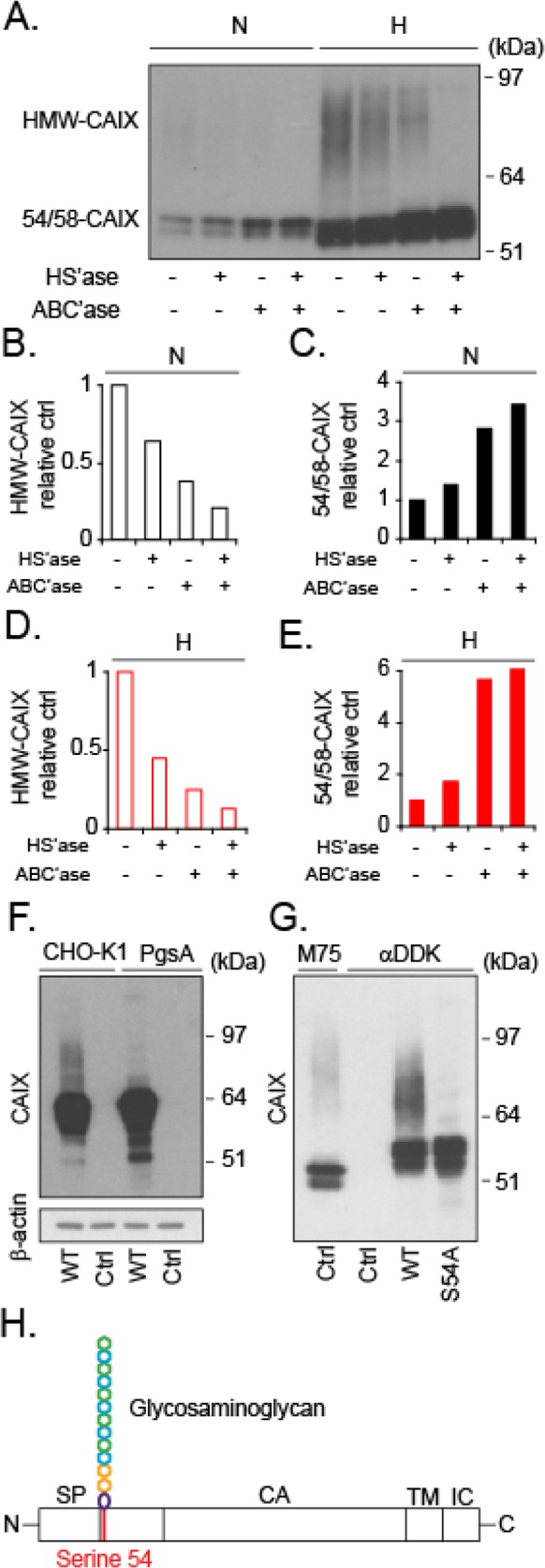
Evidence of glycosaminoglycan modification with CS or HS at serine 54 of CAIX **(A)**, Cell lysates from normoxic (N) or hypoxic (H) U87-MG cells were submitted to anion exchange chromatography and were then either untreated or digested with heparinase III (HS’ase) and/or chondroitinase ABC lyase (ABC’ase), as indicated. The digest products were separated by SDS PAGE, and CAIX was visualized by immunoblotting. **(B-E)** Densitometry measurements of high molecular weight CAIX (HMW-CAIX) **(B** and **D)** and 54/58-CAIX **(C** and **E)** in normoxic **(B** and **C)** and hypoxic cells **(D** and **E)**, from experiment shown in **(A)**. Quantitative data are related to the corresponding untreated HMW-CAIX and 54/58-CAIX, respectively (set to 1). **(F)**, Wild-type (CHO-K1) and mutant PG-deficient (PgsA) CHO cells were transfected with plasmid encoding human wild-type CAIX (WT), and analyzed for CAIX expression by immunoblotting with α-CAIX (M75) that does not cross-react with endogenous hamster CAIX, as shown by the absence of signal in untransfected cells (Ctrl). **(G)**, Wild-type (WT) plasmid encoding CAIX fused with an DDK-tag (FLAG-tag) was modified by exchanging serine 54 into alanine by site directed mutagenesis (S54A-CAIX plasmid). U87-MG cells were transfected with either the WT or S54A-CAIX plasmid (S54A) and CAIX was captured by anti-DDK antibody immunoprecipitation, as indicated. Untransfected cells (Ctrl) were subjected to M75 or anti-DDK immunoprecipitation to visualize endogenous CAIX and to exclude unspecific binding to the anti-DDK antibody, respectively. In all cases, lysates were analyzed for CAIX by immunoblotting with the M75 antibody. **(H)**, Schematic representation of CAIX, showing the position of CS or HS GAG attachment at serine 54 (red line) in relation to the domain organization of CAIX; signal peptide (SP); catalytic domain (CA); intracellular domain (IC); and transmembrane domain (TM). **(A, F)**, and **(G)** Data shown are representative of at least two independent experiments.

Using the well-established anti-CAIX antibody (α-CAIX) M75 that recognizes the CAIX extracellular domain [11], we could initially confirm that CAIX closely overlaps with the hypoxia marker GLUT1 [12] in patient glioblastoma tumors (Figure 1A), and that CAIX is substantially induced by hypoxia in a patient derived glioma cell-line (U87-MG) *in vitro* (Figure 1B). Under these conditions, we observed a dominating pool of intracellular CAIX, which should enable rapid replenishing of membrane CAIX needed for adaptation to acidic stress conditions (Figure 1B, lower panel). Further, high CAIX expression was associated with significantly worse prognosis in glioma patients (data retrieved from the REMBRANDT (Repository of Molecular Brain Neoplasia Data, NCI; Figure 1C). Immunoblotting showed CAIX at the reported 54 and 58 kDa positions [13] (hereafter referred to as 54/58-CAIX; Figure 1D). Interestingly, we also observed a previously unknown, high molecular weight (HMW) component of 70-100 kDa in U87-MG cells (Figure 1D) and in an additional cell-line derived from human glioma (Figure 1E). Like 54/58-CAIX, HMW-CAIX showed hypoxic induction and decreased but maintained expression for as long as 48 h of reoxygenation (Supplementary Figure 1A), indicating a similar half-life. CAIX has previously been shown to carry *N*-linked high mannose-type oligosaccharide and an *O-*linked di-, tri-, or tetrasaccharide [14] that could not explain the approximate 20-50 kDa size difference between CAIX variants. Proteoglycans (PGs) are proteins conjugated with another type of glycosylation, *i.e.* GAG polysaccharide chains, such as chondroitin and heparan sulfate (CS; HS) typically in the 20-50 kDa size range [15, 16]. HMW-CAIX could indeed be isolated by anion exchange chromatography commonly used for PG purification [17] (Figure 2A), and was sensitive to enzymatic digestion of GAGs; combined HS and CS digestion completely abolished HMW-CAIX concomitantly with increased 54/58-CAIX (Figure 2A–2E). Notably, there was no apparent difference in the relative levels of CS and HS between normoxic and hypoxic conditions (*cf.* Figure 2B and 2D). We next utilized parental Chinese hamster ovary (CHO)-K1 cells, and mutant CHO cells (PgsA-745) virtually devoid of GAG biosynthesis [18]. Parental CHO cells displayed the HMW-CAIX variant when transfected with a wild-type CAIX-expressing plasmid (WT-CAIX) while absent in the transfected PG-deficient cells (Figure 2F). Moreover, experiments with another CHO cell mutant (PgsD-677) that is selectively deficient in HS biosynthesis and only produces CSPG [18], indeed showed the presence of the HMW-CAIX variant, but at a lower level as compared with parental cells (Supplementary Figure 1B). We identified serine 54 of CAIX as a unique consensus serine-glycine sequence of GAG conjugation [19], and serine at this position was exchanged for alanine by site-directed mutagenesis (S54A-CAIX). Importantly, ectopic expression of the S54A-CAIX mutant completely failed to generate HMW-CAIX (Figure 2G). Together, these data provide evidence that CAIX can exist either with or without GAG modification, thus defining HMW-CAIX as a hypoxia-regulated PG, hereafter designated PG-CAIX (Figure 2H).

### Glycosaminoglycan modification regulates CAIX internalization

**Figure 3:**
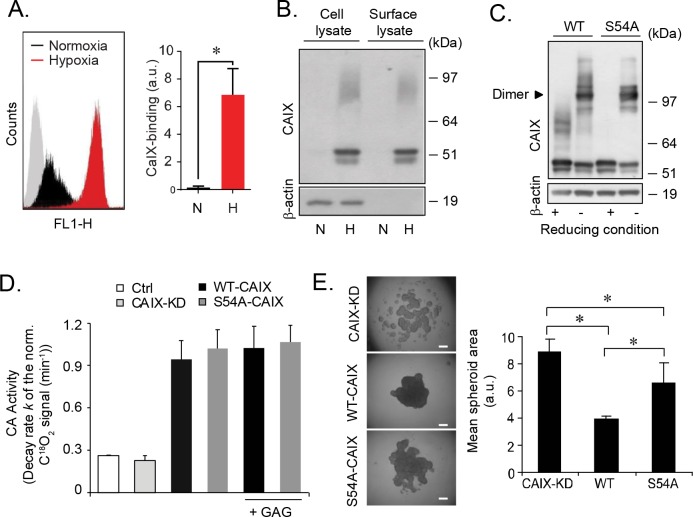
GAG substitution of CAIX regulates cell aggregation by mechanisms independent on the dimerization or catalytic activity of CAIX **(A)**, FACS quantification of cell-surface CAIX in U87-MG cells grown at normoxic (N) or hypoxic (H) conditions for 48 h. Left panel: Representative histograms; grey area shows control cells without antibody labelling. Right panel: Quantitative analysis presented as the mean ± S.D. from two independent experiments each performed in triplicates. *p<0.05. **(B)**, U87-MG cells were grown under normoxic or hypoxic conditions for 48 h followed by cell-surface biotinylation as described in Methods. Total cell lysate and biotinylated surface proteins were analyzed for CAIX and β-actin by immunoblotting. **(C)**, No role of GAG conjugation in CAIX dimerization: WT-CAIX and S54A-CAIX cell lysates were analyzed for CAIX by immunoblotting at reducing and non-reducing conditions with β-actin as loading control. **(D)**, Equal amounts of CAIX-KD, WT and S54A-CAIX cell lysates or PBS (Ctrl) were analyzed for CA activity in the absence or presence of 100 μg/mL GAG by membrane-inlet mass spectrometry analysis. Data are presented as the mean ± SD from two independent experiments. **(E)**, U87-MG CAIX-KD, WT-CAIX and S54A-CAIX cell aggregation at hypoxia. Left panel: Representative images from three independent experiments. Right panel: Quantification of cell aggregate area, which is inversely related to 3D structure forming capacity, using ImageJ. Data are presented as the mean ± SD. *p<0.05.

In order to catalyze extracellular hydration and to acidify the extracellular environment CAIX needs to be transported to the plasma membrane, and cell-surface CAIX expression was substantially increased in hypoxic as compared with normoxic cells (Figure 3A). To investigate if GAG substitution regulates CAIX cell-surface sorting, plasma membrane proteins were biotinylated and isolated by streptavidin precipitation. We found comparable ratios of 54/58-CAIX and PG-CAIX in total cell lysate and the cell-surface fraction, suggesting that constitutive transfer of CAIX to the surface does not require GAG substitution (Figure 3B). We hypothesized that GAG conjugation could facilitate CAIX dimerization required for its catalytic activity [20], as further supported by direct GAG-CAIX protein interaction (Supplementary Figure 2); both 54/58-CAIX and PG-CAIX was captured by heparin (Supplementary Figure 2A), and elution with 2 M NaCl was required to efficiently release CAIX from heparin (Supplementary Figure 2B and 2C), while CS could not prevent CAIX binding to the heparin beads (Supplementary Figure 2D). To this end, we next generated stable transfectants from CAIX knock down cells (CAIX-KD; Supplementary Figure 3A) expressing comparable levels of WT-CAIX and S54A-CAIX (referred to as WT-CAIX and S54A-CAIX cells; Supplementary Figure 3B). At odds with a role of GAG in CAIX dimerization, we found no apparent difference between WT-CAIX and S54A-CAIX dimerization (Figure 3C). Moreover, using membrane inlet mass spectrometry (MIMS) [21], we found comparable CA enzymatic activity in WT-CAIX and S54A-CAIX cells, and the addition of exogenous GAG had no effect on CA activity (Figure 3D). Also, in hypoxic CAIX-KD monolayer cultures the rescue effect on cell proliferation of S54A-CAIX was similar to that of WT-CAIX (Supplementary Figure 4A). PGs are key components in cancer cell-cell and cell-matrix adhesion [15, 16] and previous findings have suggested a supportive role of CAIX in the assembly of multicellular structures [22]. Interestingly, the reduced capacity of CAIX-KD cells to form tight 3D cell aggregates was efficiently rescued by WT-CAIX expression, whereas S54A-CAIX expressing cells produced significantly less organized 3D structures (Figure 3E and Supplementary Figure 4B). Collectively, these data indicated that GAG substitution of CAIX stimulates cell aggregation by mechanisms unrelated to the dimerization or catalytic activity of CAIX.

**Figure 4:**
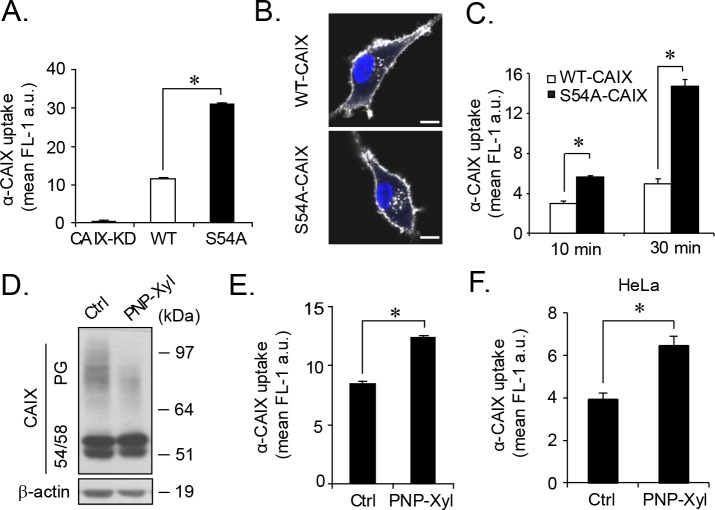
Increased CAIX internalization by genetic or pharmacological depletion of glycosaminoglycan modification **(A)**, FACS quantification of α-CAIX internalization continuously for 1 h in normoxic CAIX-KD, WT-CAIX and S54A-CAIX cells. **(B)**, Representative confocal microscopy images of WT-CAIX and S54A-CAIX cells from experiment described in **(A)**. Scale bar, 10 μm. **(C)**, α-CAIX was bound to the surface of normoxic WT-CAIX and S54A-CAIX cells on ice, followed by extensive washing, and α-CAIX internalization for 10 and 30 min at 37°C was assessed by FACS. **(D)**, U87-MG cells were either untreated (Ctrl) or treated with PNP-Xyl for 48 h in hypoxia, and cell lysates were analyzed for CAIX and β-actin expression by immunoblotting. **(E)**, U87-MG cells were treated as in **(D)** followed by α-CAIX internalization for 1 h, as evaluated by FACS analysis. **(F)**, Similar experiment as in **(E)** was performed with HeLa cells. Data are presented as the mean ± SD from three **(A, E)** or two **(C, F)** independent experiments, each performed in triplicates. *p<0.05.

Cell-cell interaction and adhesion depend on the turnover rate of cell-surface receptors, such as integrins and FGFR [23], and cell adhesion complexes are known to inhibit internalization [24]. Together with the previously described role of CAIX in cell adhesion [25] and its ability to internalize by endocytosis [26], we hypothesized that the relative deficiency of S54A-CAIX cells to form cellular aggregates (Figure 3E and Supplementary Figure 4B) was mechanistically linked to differential internalization. Strikingly, we found a substantial increase of α-CAIX uptake (approximately 3-fold) in S54A-CAIX as compared with WT-CAIX cells (Figure 4A and 4B). This was true over a wide range of α-CAIX titers (Supplementary Figure 5A) as well as for shorter and longer periods of internalization (Supplementary Figure 5B). The same results were obtained with cells pre-incubated with antibody on ice to preclude internalization, and then allowed to internalize α-CAIX for a brief time period at 37°C (Figure 4C), indicating that increased internalization in S54A-CAIX cells was not due to secondary signaling events. We found comparable levels of CAIX variants in the cell medium (Supplementary Figure 5C), suggesting that constitutive plasma membrane shedding of CAIX is not regulated by GAG substitution. Moreover, transferrin and dextran uptake through clathrin-mediated endocytosis and macropinocytosis, respectively, were not significantly different (Supplementary Figure 5D and 5E), and global cell-surface protein expression (Supplementary Figure 5F) as well as internalization (Supplementary Figure 5G) was similar, as determined by reversible cell-surface protein biotinylation integrated with flow cytometry-based quantification. These results exclude that our finding was due to a general increase in the endocytic activity of S54A-CAIX as compared with WT-CAIX expressing cells.

To corroborate a role of GAG in CAIX internalization, we next took a pharmacological approach to inhibit endogenous CAIX GAG modification using xylosides, *i.e.* small hydrophobic compounds that compete with GAG conjugation in the ER [27]. Treatment of hypoxic parental cells with the xyloside 4-Nitrophenyl β-D-xylopyranoside (PNP-Xyl) resulted in a specific decrease in PG-CAIX expression, while 54/58-CAIX expression remained intact (Figure 4D). Importantly, we found that α-CAIX internalization was significantly greater in PNP-Xyl treated than in untreated parental cells (Figure 4E). This finding was not restricted to glioma cells as comparable results were obtained with HeLa cells (Figure 4F). Collectively, these data provide evidence that specific post-translational modification with GAG can determine the internalizing capacity of the tumor cell-surface antigen CAIX.

### Glycosaminoglycan conjugation negatively regulates CAIX internalization through an increased association with caveolin-1 in membrane lipid rafts

**Figure 5:**
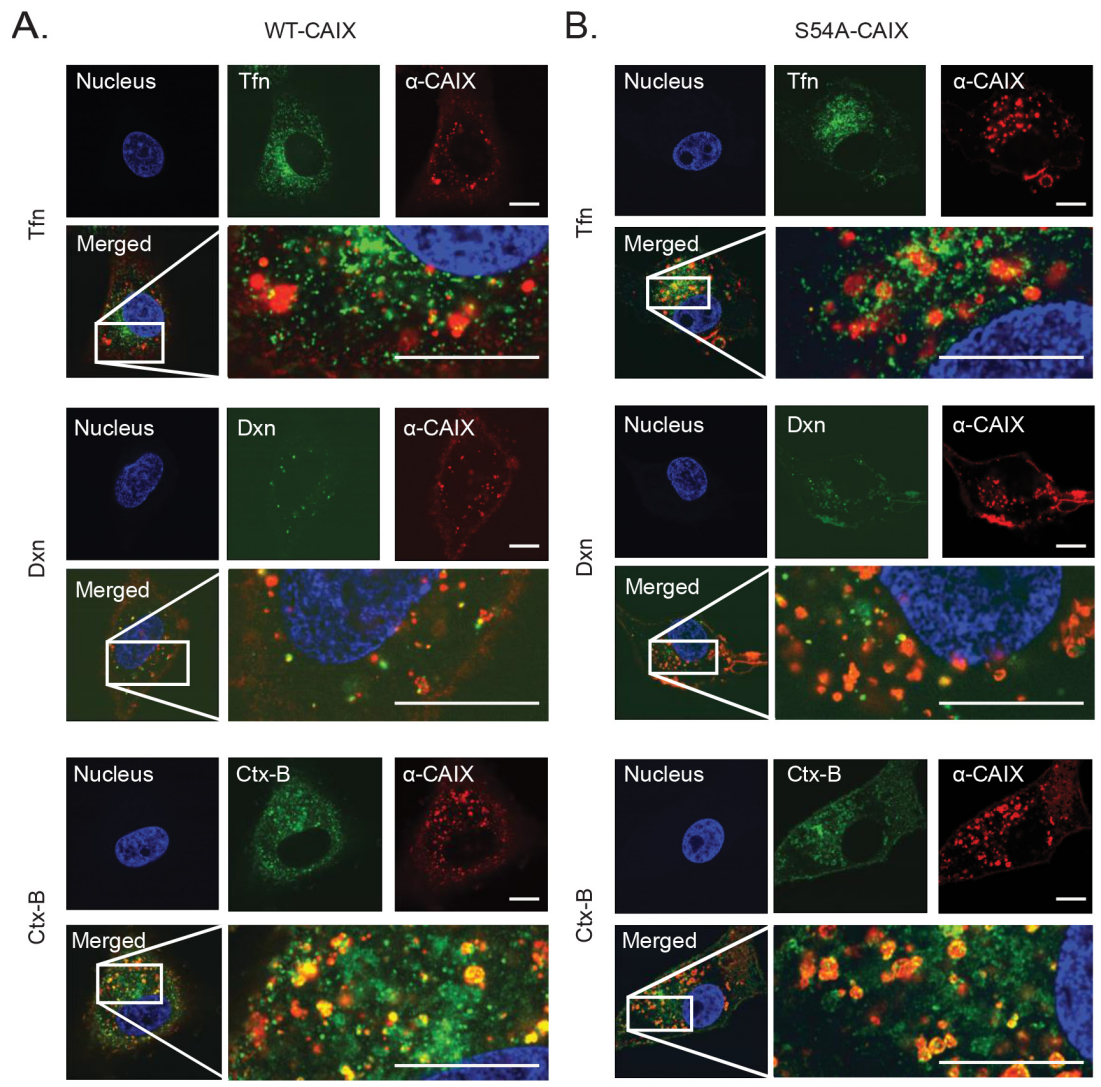
CAIX mainly follows a lipid raft-mediated endocytic pathway WT-CAIX **(A)**, and S54A-CAIX **(B)** cells were incubated with α-CAIX (red), and transferrin (Tfn; green), dextran (Dxn; green) or cholera cytotoxin subunit B (Ctx-B; green). Nuclei were counter-stained with Hoechst (blue). Merged images display strong co-localization of α-CAIX with Ctx-B (yellow signals). All images were captured on an Airyscan super resolution imaging system integrated with Zeiss 710 confocal microscope using a C-Apochromat 63X/1.20W korr M27 objective. Scale bars, 50 μM. Shown are representative images from three independent experiments, each performed in duplicate.

To elucidate the mechanisms of the inhibitory effect of GAG modification on CAIX internalization, we performed super resolution fluorescence microscopy to follow the uptake of α-CAIX in WT-CAIX and S54A-CAIX cells and its association with major endocytic pathways. We found virtually no co-localization of α-CAIX with classical, clathrin-associated endocytosis and only limited co-localization with macropinocytosis, as defined by transferrin and dextran, respectively (Figure 5A and 5B). However, there was a close overlap between α-CAIX and Cholera cytotoxin-B (Ctx-B), *i.e.* an established marker of lipid raft mediated endocytosis, both in WT-CAIX and S54A-CAIX cells (Figure 5A and 5B). Similar results were obtained in hypoxic, parental cells where internalized α-CAIX co-localized with Ctx-B (Supplementary Figure 6A). We conclude that irrespective of GAG modification, CAIX internalization mainly follows a lipid raft-associated endocytic pathway.

**Figure 6:**
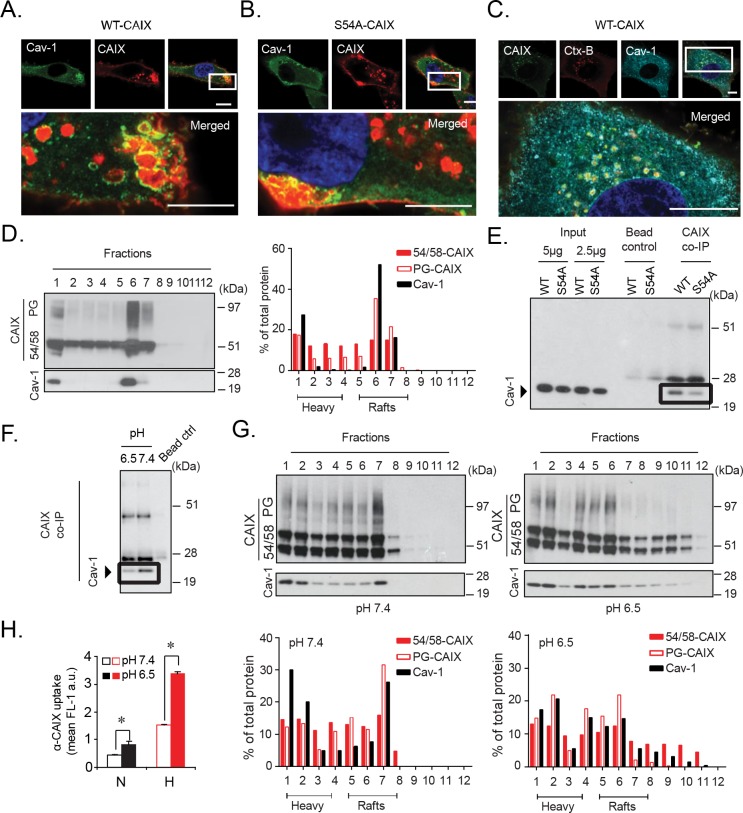
Glycosaminoglycan modification attenuates CAIX internalization through an increased association with caveolin-1 in acidosis-sensitive, lipid raft domains WT-CAIX **(A)**, and S54A-CAIX **(B)** cells were incubated with α-CAIX (red), and then stained for caveolin-1 (Cav-1; green) and nuclei (blue). In WT-CAIX cells, merged images display localization of α-CAIX mainly within caveolin-1 positive structures. **(C)**, WT-CAIX cells were co-incubated with α-CAIX (green) and Ctx-B (red), and then stained for caveolin-1 (turquoise) and nuclei (blue). Merged image shows α-CAIX and Ctx-B co-localization within caveolin-1 positive structures. Images were captured by super resolution fluorescence microscopy. **(D)**, Hypoxic parental U87-MG cells were subjected to subcellular sucrose density ultracentrifugation, followed by immunoblotting for CAIX and caveolin-1 in membrane density fractions. Right panel: Densitometry of caveolin-1, PG-CAIX, and 54/58-CAIX expressed as % of total protein in all fractions. “Heavy” and “Rafts” refer to high density (non-raft) and low density (raft) membrane fractions. **(E)**, Immunoblotting for caveolin-1 in WT-CAIX and S54A-CAIX total cell lysates (input) or following immunoprecipitation with α-CAIX (CAIX co-IP; equal protein loading). Bead control is without primary α-CAIX. **(F)**, Similar experiment as in (E) with WT-CAIX cells pre-incubated for 3 h at neutral (pH 7.4) or acidic (pH 6.5) conditions. **(G)**, Hypoxic parental U87-MG cells were incubated at neutral (left panels) or acidic (right panels) conditions, and subjected to subcellular fractionation, immunoblotting, and densitometry measurements of caveolin-1, PG-CAIX, and 54/58-CAIX (lower panels) as in (D). Microscopy images and immunoblots are representative of at least three independent experiments. Scale bars, 10 μM. **(H)**, FACS quantification of α-CAIX internalization in normoxic (N) and hypoxic (H) parental U87-MG cells following pre-treatment at pH 7.4 or 6.5. Data are presented as the mean ± SD. *p<0.05.

Caveolin-1 is a structural protein with a preference for cholesterol-rich, lipid raft membrane domains, and negatively regulates extracellular ligand uptake and global protein internalization through membrane stabilization [28–31]. Recent studies suggest that caveolin-1 scaffolding within membrane lipid microdomains results in decreased membrane protein mobility. PG internalization may follow caveolin-1 associated membrane raft endocytosis, and PG-dependent uptake of exosomes was negatively regulated by caveolin-1 [32]. Interestingly, we found that in WT-CAIX cells a substantial fraction of α-CAIX was internalized into larger, caveolin-1 positive clusters (Figure 6A) previously described as fused caveolae or complex rosettes [33]. However, in S54A-CAIX cells internalized α-CAIX was clearly less associated with caveolin-1 clusters (Figure 6B). We could further show that α-CAIX and Ctx-B co-localized in caveolin-1 positive structures in WT-CAIX cells (Figure 6C), and that CAIX co-localized with caveolin-1 in glioblastoma tumors (Supplementary Figure 6B). As expected, caveolin-1 was enriched in lipid raft membrane domains with relatively low density, as shown by sucrose gradient membrane fractionation studies (Figure 6D). Importantly, and in support of our imaging data, GAG-modified CAIX was found to strongly co-distribute with caveolin-1 in low density fractions, whereas 54/58-CAIX was more evenly distributed (Figure 6D). Moreover, co-immunoprecipitation of caveolin-1 with CAIX was greater in WT-CAIX as compared S54A-CAIX cells (Figure 6E). Collectively, these data suggest that CAIX internalization is negatively regulated by GAG modification through an increased association with membrane lipid rafts stabilized by caveolin-1.

In further support of this notion, we found that cellular acidosis, *i.e.* another common stress phenomenon of aggressive tumors [34] attenuated the direct association of CAIX with caveolin-1 (Figure 6F), which was accompanied by decreased CAIX co-localization with caveolin-1 in WT-CAIX and hypoxic parental cells (Supplementary Figure 6C and 6D) as well as re-distribution of PG-CAIX from low density membrane fractions (Figure 6G). Importantly, acidosis-induced release of PG-CAIX from caveolin-1 was associated with increased α-CAIX internalization by parental cells (Figure 6H). Moreover, the stimulatory effect of acidosis on CAIX internalization was relatively greater in WT-CAIX compared to S54A-CAIX cells at all time-points tested (uptake ratios acidosis/neutral pH: 2.5 and 1.7, respectively; Supplementary Figure 6E). These results indicate that GAG modification has a significant role in CAIX internalization also at acidic conditions, although partly attenuated by acidosis-induced redistribution of CAIX from caveolin-1 clusters in membrane rafts.

### Antibody-mediated targeting of hypoxic cancer cells is negatively regulated by glycosaminoglycan modification

**Figure 7:**
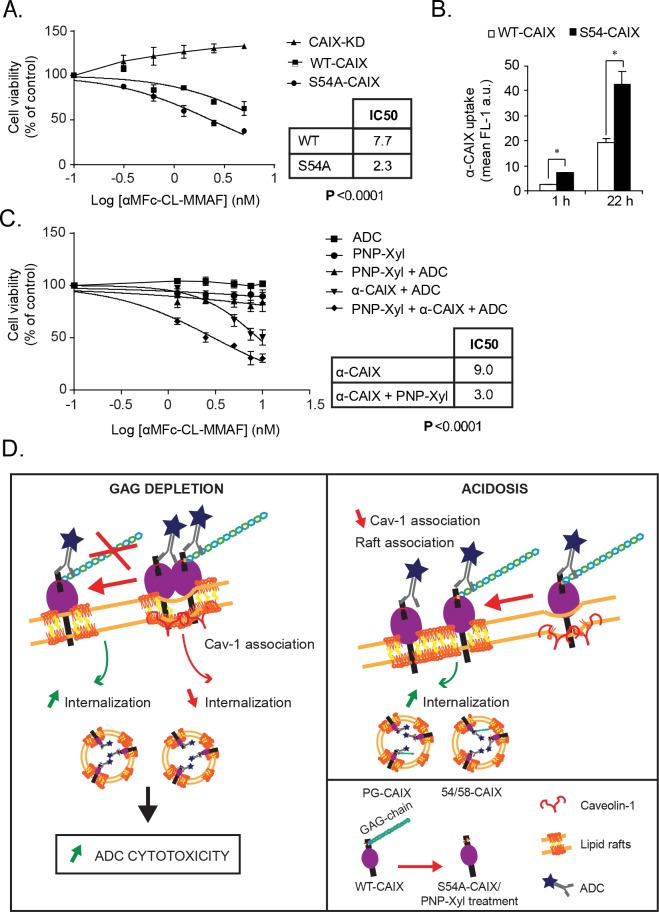
Glycosaminoglycan depletion potentiates the cytotoxic activity of CAIX-directed ADC treatment **(A)**, CAIX-KD, WT-CAIX and S54-CAIX U87-MG cells were treated with α-CAIX pre-complexed with toxin-conjugated IgG (ADC) for 72 h and assessed for cell viability by the MTS assay. **(B)**, FACS quantification of α-CAIX internalization for 1 and 22 h in WT-CAIX and S54A-CAIX cells. Data are presented as the mean ± SD. **(C)**, Hypoxic parental U87-MG cells where subjected to the various treatments, as indicated, for 72 h and assessed for cell viability by the MTS assay. ADC: Toxin-conjugated IgG only (αMFc-CL-MMAF: Fab fragment of anti-mouse IgG Fc specific antibody conjugated to monomethyl auristatin F (MMAF)); α-CAIX + ADC: α-CAIX complexed with ADC. **(A, C)** Data shown are the mean of three independent experiments ± SD. IC50 values were compared using the extra sum of squares F-test using GraphPad PRISM. **(D)**, Schematic figure of the main results of the study. Left panel: GAG depletion results in decreased CAIX association with caveolin-1 positive raft regions and increased internalization, which translates into enhanced α-CAIX-ADC cytotoxicity. Right panel: Acidic conditions reduce CAIX association with caveolin-1 in raft domains, resulting in increased CAIX internalization.

Our finding that GAG modification negatively regulates CAIX internalization may have direct implications for antibody-based targeting of hypoxic cancer cells. We next set out to explore this possibility by complexing α-CAIX with an antibody conjugated with monomethyl auristatin F, *i.e.* an antimitotic agent under clinical development for ADC-based treatment of cancer [35]. The target specificity of the α-CAIX-ADC complex was shown by the complete resistance of CAIX-KD cells (Figure 7A). Consistent with an inhibitory role of GAG in ADC delivery, we found that α-CAIX-ADC-mediated targeting was more efficient in S54A-CAIX as compared with WT-CAIX cells with IC50 values of approximately 2.3 and 7.7 nM, respectively (Figure 7A). We could confirm that under these conditions of long-term α-CAIX incubation (approximately 22 h) the increased uptake in S54A-CAIX compared to WT-CAIX cells remained (Figure 7B). Interestingly, combined treatment of hypoxic parental cells with PNP-Xyl to pharmacologically inhibit protein GAG modification and thereby increase CAIX internalization (Figure 4D–4F), resulted in increased cell killing efficiency of α-CAIX-ADC (IC50 values of 3.0 and 9.0 nM with and without PNP-Xyl, respectively; Figure 7C). We conclude that genetic or pharmacological inhibition of CAIX GAG modification results in increased CAIX internalization, and potentiates the cytotoxic activity of CAIX-directed ADC treatment.

## DISCUSSION

Post-translational modification by glycosylation provides greater diversity than any other modifications of the proteome, and altered protein glycosylation patterns emerge as a new generation of diagnostic biomarkers in cancer [36]. Further, glycosylation-targeting antibodies are being developed for a broad range of cancers, based on the recognition of glycan epitopes aberrantly expressed in malignant cells [37, 38]. In this study, we provide evidence of a role for glycosylation in tumor antigen internalization with potential implications for ADC delivery and cytotoxic activity in cancer cells. Our results thus introduce another level of regulation by glycosylation that may have general relevance for targeted drug delivery strategies in cancer.

We find that decreased CAIX internalization by GAG modification is not related to GAG-dependent regulation of CAIX dimerization or catalytic activity. Instead, PG-CAIX showed increased association with caveolin-1 clusters in lipid raft domains. Caveolin-1 is a major structural component of lipid rafts with a preference for cholesterol-rich membrane regions where it negatively regulates extracellular ligand uptake and global protein internalization through membrane stabilization [28–33]. Our data thus suggest that membrane raft endocytosis has a significant role in CAIX internalization, and that the inhibitory effect of GAG conjugation is linked to an increased sorting of CAIX to raft domains enriched in caveolin-1. Accordingly, we show that acidosis-induced internalization of CAIX is associated with a partial relocation of PG-CAIX from raft regions along with caveolin-1 (see Figure 7D). Since CAIX protein levels are expected to be highly induced at acidic conditions in conjunction with hypoxia, these data point at an important role of CAIX GAG modification under stress conditions typically found in aggressive tumors.

PGs, *i.e.* proteins modified by GAGs, constitute a family of highly polyanionic macromolecules found at the cell-surface as well as in the surrounding matrix. The multifaceted role of PGs in tumor development is well-documented, and is in many cases mediated through PG-dependent endocytosis. Differential modes of membrane attachment of cell-surface PGs may preferentially sort them either to clathrin-coated or lipid raft plasma membrane domains. Numerous GAG-binding ligands utilize lipid raft-mediated pathways, including caveolin-1 associated endocytosis [15]. Our finding that GAG modification inhibits CAIX endocytosis is thus unexpected in relation to the established role of PGs as pleiotropic endocytosis receptors of *e.g.* lipoproteins, exosomes, viruses and peptide-nucleic acid complexes [15]. Cell-surface PGs may interact with ligands with specificity for the GAG or the core protein part, and depending on the relative abundance of the two classes of ligands the GAG chain can have both positive and negative effects on ligand internalization. How GAG modification regulates the mobility of membrane proteins and further cargo internalization in a more complex ligand environment should be an interesting area of future investigations.

We have identified CAIX as a part-time PG that is optionally substituted with GAG of either the CS or HS type. Interestingly, several cell-surface proteins have been found to appear with or without GAG substitution, *i.e.* the hypoxia-induced stem cell marker and potential ADC target CD44 [39], and thrombomodulin [40] where GAG modification regulates cell signaling activation and anticoagulation activity, respectively. Thus, an increased understanding of the mechanisms that regulate GAG modification of CAIX and other tumor antigens should contribute to improved strategies for cancer cell targeting. Moreover, CAIX also carries *N*-linked high mannose-type oligosaccharide and an *O-*linked di-, tri-, or tetrasaccharide [14], and future studies should address their potential relevance in CAIX localization and internalization.

Our findings highlight the important regulation of tumor antigen internalization by glycosylation. This motivates increased consideration of strategies to deplete target protein glycosylation, *i.e.* by the use of functional antibodies for glycosylation editing [41] to optimize the efficiency of ADCs and other drug-conjugate treatments. Further, aberrant expression of key modulating enzymes in cancer cells may generate changes in glycan epitopes that could be exploited for further fine-tuning of ADC target specificity. We conclude that analyses of the pattern and extent of tumor cell glycosylation deserves increased attention in the future development and prediction of cancer treatments targeted at cell-surface tumor antigens.

## MATERIALS AND METHODS

Descriptions of S54A-CAIX plasmid construction, immunofluorescence imaging, membrane protein biotinylation and endocytosis, endocytic ligand uptake, immunoblotting, immunoprecipitation and heparin binding, and membrane inlet mass spectrometry experiments are provided in Supplementary Information.

### Materials

Heparinase III (H8891), chondroitinase ABC (C2905), 4-Nitrophenyl β-D-xylopyranoside (PNP-Xyl; N2132), CS-4 sodium salt from bovine trachea (C9819), heparin, biotin-HRP (S3438), heparin-agarose (H0402), G-418 disulfate salt (A 8601), puromycin (P9620), *Ca9* shRNA MISSION lentiviral transduction particle (SHCLNV; TRCN0000150123), MISSION pLKO.1-puro Non-Mammalian shRNA Control Transduction Particles (SHC002V), iodoacetamide, and FITC-conjugated 10 kDa dextran were from Sigma-Aldrich. Anti-β-actin (ab8227), anti-GLUT-1 (ab32551) and anti-Caveolin-1 (ab2910) antibodies were from Abcam. M75 anti-CAIX antibody (α-CAIX) was the same as previously described [13, 26, 42, 43], and is commercially available from BioScience, Slovakia. Alexa Fluor (AF)-conjugated secondary IgG antibodies, Transferrin-AF488 (T13342), streptavidin-AF-488 (S32354), immunoprecipitation Kit-Dynabeads® Protein G (10007D), Cholera toxin-subunit B-AF-488 (C34775), biotin EZ-Link Sulfo-NHS-SS-Biotin (21331), streptavidin Sepharose High Performance (17-5113-01), NuPage 4-12% Bis Tris gels, SeeBlue Plus2, and MesNa (sodium-2-mercaptoethanesulfonate) were from Thermo Fisher Scientific. Protein G agarose beads (sc-2002) was from Santa Cruz Biotechnology, and Myc-αDDK-tagged (FLAG-tag) ORF clone of Homo sapiens Ca9 (RC204839) and rabbit anti-αDDK antibody (TA100023) were from Origene. QuickChange II Site-directed mutagenesis Kit (200523) was from Agilent Technologies, and CellTiter 96® AQueous One Solution Cell Proliferation Assay MTS reagent from Promega. The anti-Mouse IgG drug conjugate Fc-(monomethyl auristatin F) MMAF antibody (αMFc-CL-MMAF) was from Moradec, and H_2_^18^O from Cambridge Isotope Laboratories. BCA^TM^ Protein Assay Kit was from Pierce, and complete protease inhibitor from Roche Diagnostics. All chromatography columns were from Amersham Biosciences and fine chemicals were from Sigma-Aldrich.

### Cells and clinical samples

U87-MG, U251-MG, HeLa, wild-type CHO-K1, HS-deficient pgsD-677, and PG-deficient pgsA-745 CHO cell-lines were freshly purchased from ATCC (during 2013-2016), authenticated by STR DNA profiling (ATCC) and passaged < 6 months following receipt. CHO cells were routinely cultured in F12K and other cells in DMEM medium, supplemented with 10% foetal bovine serum (FBS), 2 mM L-glutamine, 100 U/mL penicillin and 100 μg/mL streptomycin (growth medium). All cells were grown in a humidified 5% CO_2_ incubator at 37°C. For hypoxia experiments, cells were incubated in a humidified Sci-tive NN Hypoxia workstation (Ruskinn Technology) set at 5% CO_2_, 1% O_2_, and 37°C. For acidosis experiments, cells were grown in DMEM adjusted to the indicated pH levels.

U87-MG cells were transformed with Non-Mammalian Control shRNA (Ctrl) and *Ca9* shRNA (CAIX-KD) lentiviral transduction particle according to the manufacturer's instructions and were routinely cultured in growth medium supplemented with puromycin. CAIX-KD U87-MG cells were transfected with WT-CAIX or GAG-deficient CAIX plasmid (S54A-CAIX) by electroporation (ECM 399, BTX Harvard Apparatus) followed by selection for neomycin resistance. To isolate stably transfected subclones with equal CAIX expression, WT-CAIX and S54A-CAIX cells were incubated with M75 α-CAIX on ice for 30 min, washed in PBS/1% BSA and labelled with AF488-anti-mouse antibody on ice for 30 min and again washed in PBS/1% BSA. WT and S54A-CAIX cells were sorted according to equal fluorescence signal intensity using a FACS Aria (BD) into a collection buffer containing 50% selection media (12 μg/mL puromycin, 2 mg/mL G418, 1% PEST and 10% FBS) and 50% FBS, and were then expanded for assessment of CAIX expression by Western blotting.

Tumor specimens were obtained from patients with primary glioblastoma (World Health Organization grade IV) from the Department of Neurosurgery, Skåne University Hospital, Lund. Biopsies were collected with informed consent according to Protocol H15 642/2008 approved by the Lund University Regional Ethics Board, Lund, Sweden.

### FACS quantification

Surface labelling of CAIX was performed by incubating detached cells with M75 α-CAIX for 30 min at 4°C, and then for 30 min with AF-488-conjugated secondary antibody in 1% BSA/PBS. For detection of CAIX internalization, M75 antibody (1/200) was pre-complexed with AF-488 secondary antibody (1/500) in serum free (SF) medium for 30 min, and then added to adherent cells during the indicated time periods at 37°C. In some cases, antibody complexes were pre-bound to cells on ice, followed by extensive washing with PBS, and internalization at 37°C for 10 or 30 min. To inhibit CAIX GAG modification pharmacologically, parental cells were pre-treated in DMEM supplemented with 2.5 mM PNP-Xyl for 48 h prior to FACS analysis. Data were acquired on an Accuri C6 flow cytometer and analyzed using Accuri C6 software (BD Biosciences). Data are expressed as arbitrary units (a.u.), which represents mean cellular fluorescence corrected for autofluorescence background signal as quantified in cells incubated without antibody or antibody-complexes.

### Confocal microscopy and super resolution microscopy

Cells were fixed in 4% (w/v) paraformaldehyde for 10 min on ice, and permeabilized with 0.5% Triton-X-100 in PBS for 10 min on ice. Cells were stained with α-CAIX (M75) (1/200) and rabbit anti-Caveolin-1 antibody (1/100), and then with AF-488 or -546-conjugated secondary antibodies (1/300) for 1 h at RT. Cell nuclei were counterstained with Hoechst. For CAIX internalization studies, cells were incubated with α-CAIX pre-complexed with AF-488 or AF-546 conjugated secondary antibody for 1 h followed by washing with 1 M NaCl in PBS and PBS to remove remaining surface bound complexes prior to fixation. For co-internalization studies, cells were co-incubated with α-CAIX antibody complex, and either Transferrin-AF488 (100 μg/mL), Cholera toxin B-AF488 (10 μg/mL) or Dextran-FITC (1000 μg/mL) under the same conditions. Cells were analyzed using Zeiss LSM 710 confocal scanning equipment with a Plan-Apochromat20x/0.8 objective or a C-Apochromat 63x/1.2 W Korr objective and Zen software. Super resolution imaging details of the endocytic routes were acquired using the Airyscan detector system (Zeiss).

### Cell membrane fractionation

Subcellular membrane fractionation was performed using a modified detergent-free method [44]. Hypoxic cells were grown in pH 7.4 or 6.5 for the indicated time-points in serum-free medium, scraped in lysis buffer (150 mM Na_2_CO_3_, pH 11, containing 1 mM EDTA, protease inhibitor mixture), before sonication with 3 cycles of 20 s bursts (QSonica Q125 sonicator). For the gradient ultracentrifugation, 1 mL of membrane homogenate was mixed with 1 mL of 80% sucrose in 25 mM MES (2-(N-Morpholino)ethanesulfonic acid sodium salt), 150 mM NaCl (MBS, pH 6.5) to form 40% sucrose and loaded at the bottom of an ultracentrifuge tube. Discontinuous sucrose gradients (generated by layering 6 mL of 35% sucrose in MBS followed by 4 mL of 5% sucrose) were centrifuged at 175,000 g using an SW41Ti rotor (Beckman Instruments) for 3 h at 4°C. Total protein from 150 μL of each fraction was precipitated by methanol/chloroform, and the final pellet was air-dried and resuspended in 25 μL of RIPA buffer. Equal volumes of precipitated protein were loaded from each fraction for immunoblotting, and densitometry quantification as described above.

### Anion chromatography PG isolation and enzyme digestion

Cells were washed in PBS and lysed in 2% Triton X-100 buffer (0.15 M NaCl, 10 mM KH_2_PO_4_, 10 mM EDTA, 5 μg/mL ovalbumin (OVA), pH 7.5, 2% Triton X-100, 1x Complete Protease Inhibitor) for 30 min at RT. PGs were isolated by anion exchange chromatography using DEAE-cellulose as previously described [17]. Isolated PGs were resuspended in digestion buffer (50 mM NaOAc, 150 mM NaCl, 5 mM CaCl_2_, 50 mM HEPES-HCl, pH 6.5, 0.5x complete protease inhibitor without EDTA) followed by incubation with or without 0.6 mIU/mL heparinase III and/or 60 mU/mL chondroitin ABC lyase at 37°C O/N and then processed further for immunoblotting analysis.

### Antibody drug conjugate cytotoxicity assay

U87-MG WT-CAIX, U87 S54A-CAIX, and U87 CAIX-KD cells (3,000 cells/well) were seeded in 96-well plates and incubated O/N at 37°C. On the following day, α-CAIX (1/300) with or without the addition of various concentrations (0.3125-5 nM) of αMFc-CL-MMAF were added in SF medium. Alternatively, U87 WT-CAIX cells (2,500 cells/well) following O/N incubation were treated with or without PNP-Xyl (1 mM) for 24 h to inhibit GAG biosynthesis prior to the addition of α-CAIX with or without anti-mouse IgG Fc-MMAF (1.25-10 nM). The cells were then incubated for another 72 h, and assessed for viability using the MTS reagent by absorbance measurement at 490 nm in a VERSAmax microplate reader.

### Statistical analyses

All presented immunoblotting and imaging experiments were performed in duplicate or triplicate in at least three independent experiments unless stated otherwise. Student's t-test was employed for the comparison of groups containing three or more replicates. Statistical significance was set at p < 0.05. Error bars represent standard deviation (SD). IC50 values were compared using the extra sum of squares F-test using GraphPad PRISM.

## SUPPLEMENTARY MATERIALS FIGURES



## Abbreviations

ADC: antibody-drug conjugate
CAIX: carbonic anhydrase IX
CHO: Chinese hamster ovary
CS: chondroitin sulfate
GAG: glycosaminoglycan
HS: heparan sulfate
PG: proteoglycan
