# Supplementary material for: Tumor antigen glycosaminoglycan modification regulates antibody-drug conjugate delivery and cytotoxicity
Source: Oncotarget. 2017 Apr 7;8(40):66960–74. doi: 10.18632/oncotarget.16921 (PMC5620149; doi:10.18632/oncotarget.16921)
Supplement: Supplementary file 1 [file oncotarget-08-66960-s001.pdf]

Tumor antigen glycosaminoglycan modification regulates antibody-drug conjugate delivery and cytotoxicity

MATERIALS AND METHODS

Construction of S54A-CAIX plasmid

TheMyc-αDDK-tagged ORF clone of Homo sapiens CAIX (RC204839) plasmid was subjected to site-directed mutagenesis using the QuikChange II Site-Direct Mutagenesis Kit(Agilent Technologies), and oligonucleotide primers designed to generate a plasmid encoding an alanine in exchange for serine at position 54 (antisense: 5'-gtcatcttccccagcagagcctcctccca-3', sense: 5'-tg ggaggaggctctgctggggaagatgac-3'). Premade Z-Competent *E. coli* Cells (Zymo Research; DH5α) transformed with the WT or S54A-CAIX plasmid were selected for kanamycin resistance, and plasmids were prepared using GenElute Endotoxin-free Plasmid Maxi Kit (Sigma).

Immunofluorescence imaging of tumor sections

Human glioblastoma tumor cryosections were fixed with ice-cold 70% ethanol and blocked in PBS with Tween 20 and 5% FBS, followed by incubation overnight (O/N) at 4°C with primary antibodies to CAIX (1/100) and GLUT-1 (1/100) or caveolin-1 (1/100) in blocking buffer, and then with AF488 or -546-conjugated secondary antibodies (1/300) for 1 h at room temperature (RT). Control sections were stained with AF-conjugated secondary antibodies alone in parallel. Sections were mounted with Fluorescent Mounting Medium (Dako), and analyzed using an Axio Observer.Z1 HB 100 fluorescence microscope equipped with a 20× water objective (Carl Zeiss).

Membrane protein biotinylation and endocytosis

Cell-surface proteome biotinylation was performed as previously described [1]. Briefly, WT and S54A-CAIX cells were incubated on ice with 1 mg/mL of a membrane-impermeable and cleavable biotin moiety (EZ-Link® Sulfo- N-hydroxysuccinimide (NHS)-SS-Biotin), and free biotin was quenched with 0.1 M Glycine in Mg/Ca-PBS. Where appropriate, endocytosis was initiated by the addition of pre-warmed SF medium for 1 h at 37°C, and then stopped by incubation on ice for 10 min. For removal of cell-surface biotin, cells were incubated with 300 mM MesNa in 50 mM Tris pH 8.6 containing 100 mM NaCl, 1 mM EDTA and 0.2% BSA, rinsed with Mg/Ca-PBS and then incubated with iodoacetamide (5 mg/mL) in Mg/Ca-PBS. Surface and internalized protein biotinylation was quantified by FACS after cell detachment, fixation with 2% paraformaldehyde, permeabilisation with 0.5% saponin in PBS, and labelling with streptavidin-AF-488 (5 µg/mL) for 30 min at 4˚C. Results were expressed after subtraction of the values of negative control cells (no biotinylation + streptavidin-AF-488 labelling). The internalized protein-biotin pool was expressed as percentage of the total cell-surface protein-biotin signal after subtraction of residual biotin after MesNa treatment.

Endocytic ligand uptake

Cells were incubated with 100 µg/mL of Transferrin-Alexa-488, 5 µg/mL of Cholera-Toxin subunit B-Alexa-488 (Life Technologies), or 500 µg/mL of Dextran-FITC (Sigma Aldrich) during 30 min at 37˚C in SF medium. Cells were detached, washed and resuspended in PBS, and cell-associated fluorescence was acquired on an Accuri C6 Flow cytometer and analyzed using Accuri C6 software (BD Biosciences).

Immunoblotting

Cells were lysed with radioimmunoprecipitation assay (RIPA) buffer (10 mM Tris-HCl pH 7.4, 150 mM NaCl, 1 mM EDTA, 0.1% SDS, 1% Triton X-100, 1% sodium deoxycholate) or non-denaturing lysis buffer (20 mM Tris-HCl at pH 8.0, 137 mM NaCl, 1% Triton X-100, and 2 mM EDTA) containing Complete protease inhibitor. Samples were centrifuged at 4,500 *g* for 10 min, and supernatants were collected. Protein concentration was determined using the BCA™ Protein Assay Kit, equal amounts of proteins were mixed with 4x NuPage LDS sample buffer and reducing agent and separated by electrophoresis in a 4-12 % NuPAGE Bis-Tris gel. The proteins were transferred to PVDF membranes (Pall Corporation), and blocked with TBS 0.05% Tween 20 containing 5% milkfor 1 h at RT. Membranes were probed by incubation with the following primary antibodies O/N at 4°C: Anti-caveolin-1 ab2910 (1/4,000), α-CAIX M75 (1/200), anti-β-actin ab8227 (1/5,000), and then incubated with horseradish peroxidase conjugated anti-rabbit (1/10,000) or anti-mouse IgG (1/10,000) secondary antibodies. Protein bands were visualised by ECL western blotting substrate, and their intensities were quantified by densitometry using ImageJ software (NIH).

Immunoprecipitation and heparin binding experiments

For immunoprecipitation, cell lysates were swirled with α-CAIX or anti-αDDK antibody at 4°C O/N as indicated in the figure legends. The antibody-antigen solution was mixed with Protein G conjugated Dynabeads or Protein G agarose beads for 3 h at 4°C followed by extensive washing using a DynaMag-2 magnetic separator or centrifugation. Bound proteins were eluted according to the manufacturer's recommendation for immunoblotting analysis or stored bound to the agarose beads for CA activity measurement (described below). For heparin binding experiments, cell lysates were pre-incubated with or without heparin or CS (100 µg/mL) followed by swirling with heparin-agarose O/N. Beads were extensively washed in PBS or, for NaCl elution experiments, extensively washed in PBS followed by incubation with PBS containing 1 M or 2 M NaCl twice for 40 min at 4°C. Beads were pelleted in between washes for 5 min at 2,000g, the supernatants were collected and pooled with two consecutive NaCl washes. Remaining bound proteins were then eluted by boiling the beads in 0.1 M glycine, pH 2.5, 1x LDS and reducing agent, and collected samples were finally analyzed for bound and released (supernatants) CAIX by immunoblotting.

CAIX activity analysis by membrane-inlet mass spectrometry

Relative CA activity in WT-CAIX and S54A-CAIX cells was determined by time-resolved membrane-inlet mass spectrometry (MIMS) [2] according to the procedure described in Benlloch *et. al.* [3]. This procedure allows accurate mass spectrometric detection of isotopic exchange of ^{18}O-labelled HCO_3^- and CO_2 with the much lower enriched water in the MIMS cell. This process is detected as a change in $^{12}C^{18}O_2$ concentration after the injections of air-equilibrated ^{18}O-labelled water. The slope of the $^{12}C^{18}O_2$-signal (normalized to the sum of all CO_2 isotopologues) provides a measure of the pseudo first-order rate constant for hydration of CO_2 by the CA activity of the measured sample. The rate of the CA reaction is compared to the (non-catalyzed) rate of chemical CO_2 equilibration in the same assay solution [2]. Samples were loaded in a MIMS cell (based on the Clark-type O_2 electrode chamber with adjusted volume of 300 µl) separated from the high vacuum (3×10^{-8} bar) of the mass spectrometer *via* a 25 µm gas permeable silicon membrane supported by a porous Teflon support (Ø 1 cm). The MIMS cell was connected to an isotope ratio mass spectrometer (DELTA V Plus, Thermo Fischer Scientific) *via* a cooling trap (dry ice + EtOH). After approximately 5 min of sample incubation under continuous stirring of sample suspensions inside the MIMS cell, a stable baseline was reached, and 5 µl of air-equilibrated $H_2^{18}O$ (97% enrichment) was injected. The CA activity was monitored online as the change in $^{12}C^{18}O_2$ concentration after the injection of $H_2^{18}O$, and the signal was normalized to a sum of all CO_2 species detected as the non-labelled ($^{12}C^{16}O_2$), singly-labelled ($^{12}C^{16}O^{18}O$), and doubly-labelled ($^{12}C^{18}O_2$) isotopologues at *m/z* 44, *m/z* 46 and *m/z* 48, respectively. All measurements were performed at 20°C and pH 7.4. To avoid carryover of CA activity from one experiment to the next, the MIMS chamber was flushed with a weak HCl solution (pH of ~2) and then thoroughly washed with deionized water. MIMS signals obtained were analyzed using *OriginPro 9* software. CA activity was expressed as a rate of monoexponential decay of the normalized $^{12}C^{18}O_2$-signal, and compared to the unanalyzed rate of CO_2 equilibration obtained in the assay buffer (PBS, pH 7.4) in the absence of samples.

REFERENCES

1. Bourseau-Guilmain E, Menard JA, Lindqvist E, Indira Chandran V, Christianson HC, Cerezo Magana M, Lidfeldt J, Marko-Varga G, Welinder C, Belting M. Hypoxia regulates global membrane protein endocytosis through caveolin-1 in cancer cells. Nat Commun. 2016; 7:11371.
2. Beckmann K, Messinger J, Badger MR, Wydrzynski T, Hillier W. On-line mass spectrometry: membrane inlet sampling. Photosynth Res. 2009; 102:511-522.
3. Benlloch R, Shevela D, Hainzl T, Grundstrom C, Shutova T, Messinger J, Samuelsson G, Sauer-Eriksson AE. Crystal structure and functional characterization of photosystem II-associated carbonic anhydrase CAH3 in Chlamydomonas reinhardtii. Plant Physiol. 2015; 167:950-962.

SUPPLEMENTARY FIGURES

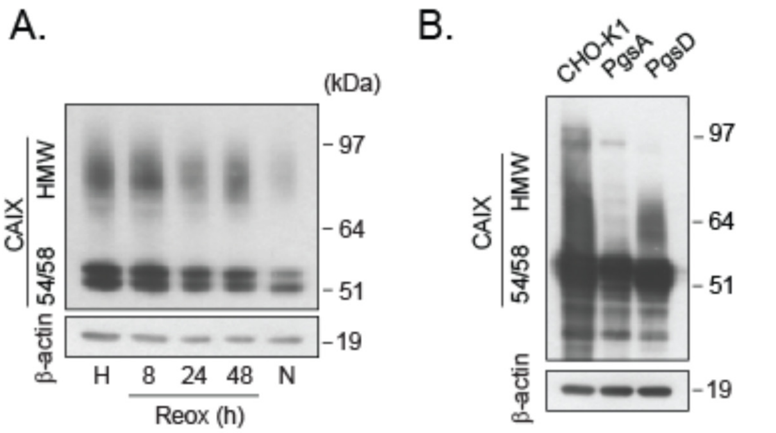

Supplementary Figure 1: Glycosaminoglycan modification of CAIX. (A), Immunoblotting for CAIXin U87-MG cell lysates grown under normoxic or hypoxic conditions for 48 h followed by re-oxygenation, as indicated, shows no apparent difference in half-life between HMW and 54/58-CAIX. **(B)**, Wild-type (CHO-K1) and mutant PG-deficient (PgsA) or HS-deficient (PgsD) CHO cells were transfected with plasmid encoding human wild-type CAIX, and analyzed for CAIX expression by immunoblotting with α-CAIX (M75). Shown are representative immunoblots from three **(A)** and two **(B)** independent experiments.

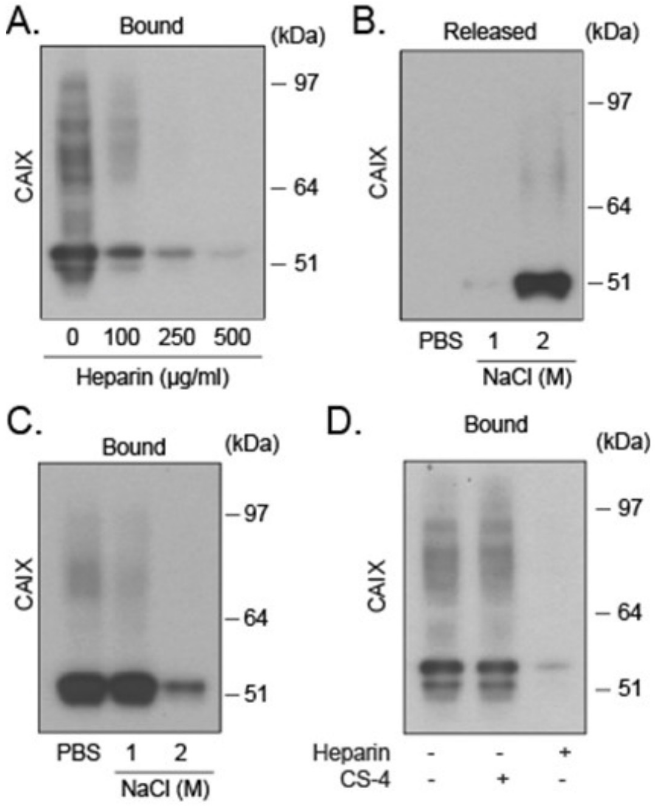

Supplementary Figure 2: CAIX protein binds to glycosaminoglycan. (A), Hypoxic U87-MG cell lysates were incubated with heparin-agarose in the absence or presence of various concentrations of free heparin, and bound CAIX was determined by immunoblotting. **(B** and **(C)**, Hypoxic U87-MG cell lysates were incubated with heparin-agarose in PBS, 1 M NaCl or 2 M NaCl, and released **(B)** and bound **(C)** CAIX was determined by immunoblotting. **(D)**, Hypoxic U87-MG cell lysates were incubated with heparin-agarose in the absence or presence of free heparin or chondroitin sulfate (CS-4), and bound CAIX was determined by immunoblotting. Shown are representative immunoblots from at least two independent experiments.

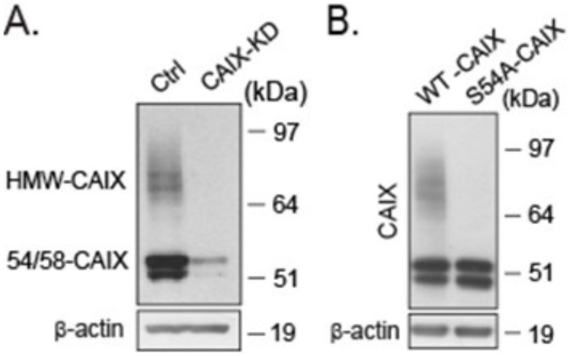

Supplementary Figure 3: CAIX expression in CA-KD, WT-CAIX, and S54A-CAIX cells. (A), U87-MG cells were stably transformed with a lentiviral vector encoding non-target control (Ctrl) short hairpin RNA (shRNA) or shRNA targeting CAIX (CAIX-KD). Efficient CAIX KD was confirmed by immunoblotting for CAIX in hypoxic cells. **(B)**, CAIX-KD cells were stably transfected with the WT-CAIX or S54A-CAIX plasmid and CAIX expression pattern was analyzed by immunoblotting. Data shown are representative of at least three independent experiments.

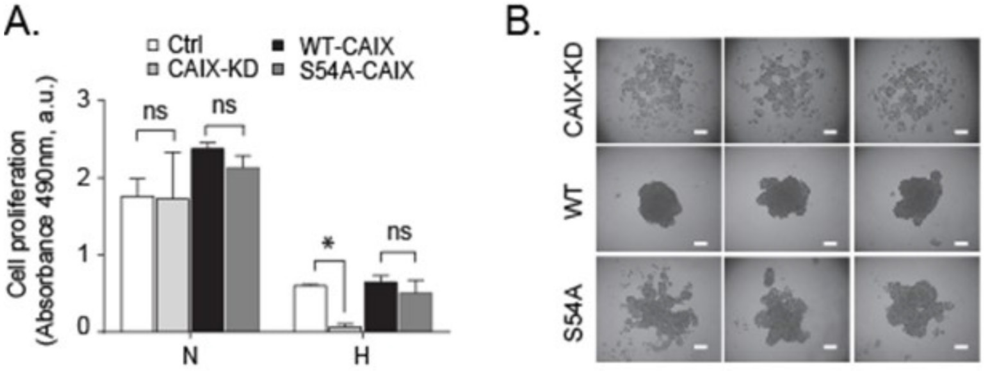

Supplementary Figure 4: Role of CAIX glycosaminoglycan modification in cell aggregation. (A), No role of CAIX GAG modification in hypoxic cell proliferation. U87-MG parental (Ctrl), CAIX-KD, WT-CAIX and S54A-CAIX cells were grown at normoxia (N) or hypoxia (H) for 72 h, and cell proliferation was assessed by the crystal violet method. Data are presented as the mean ± SD from two independent experiments, each performed in triplicates. *$p<0.05$; ns, not significant. **(B)**, U87-MG CAIX-KD, WT-CAIX and S54A-CAIX cell aggregation was analyzed by the 3D cell aggregation assay at hypoxia. Shown are three representative images from each condition.

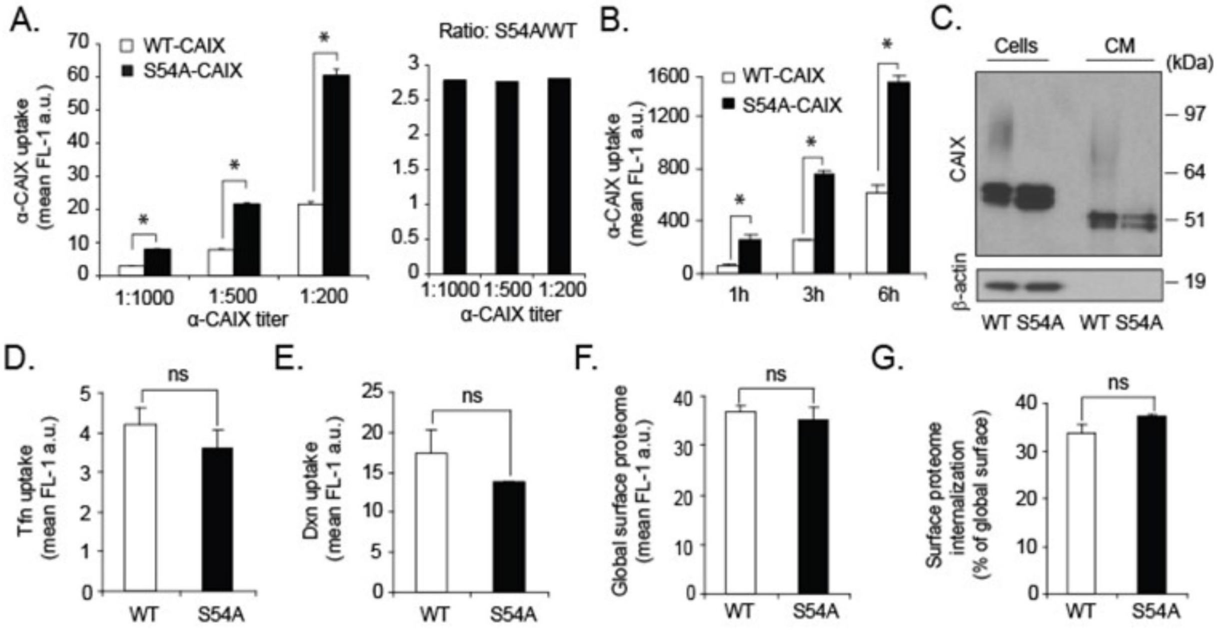

Supplementary Figure 5: Glycosaminoglycan modification negatively regulates CAIX internalization. **(A)**, FACS quantification of M75 α-CAIX internalization at the indicated titers for 1 h in WT-CAIX and S54A-CAIX cells. Right panel shows that α-CAIX internalization was approximately 2.7-fold higher in S54A-WT as compared with WT-CAIX cells at all titers tested. **(B)**, Similar experiment as in **(A)** with α-CAIX incubation at the indicated time periods, again showing a consistently greater uptake in cells expressing CAIX without GAG modification. Data are presented as the mean ± SD, each performed in triplicate. *$p<0.05$. **(C)**, No role of GAG in CAIX shedding. Conditioned media (CM) and corresponding cell lysates from CAIX-WT and S54A-CAIXcells were analyzed for CAIX by immunoblotting with β-actin as loading control. Shown are representative immunoblots from at least two independent experiments. **(D-G)**, No difference in global endocytosis between S54A-CAIX and WT-CAIX cells. S54A-CAIX and WT-CAIX cells exhibit comparable uptake of transferrin **(D)** and dextran **(E)** as well as total **(F)** and internalized **(G)** biotinylated cell-surface proteome as determined by FACS analysis. Data are presented as the mean ± SD from three independent experiments. *$p<0.05$.

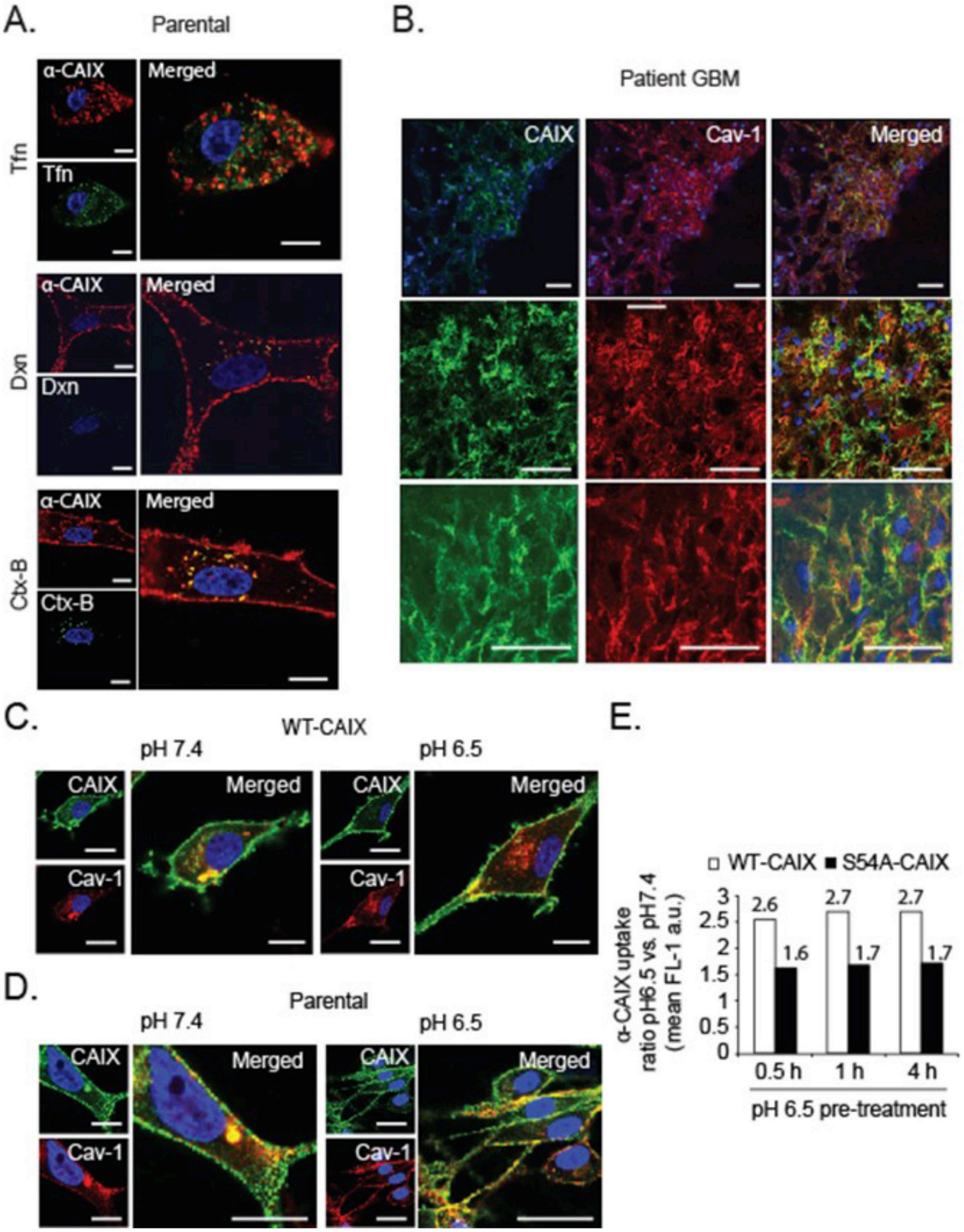

Supplementary Figure 6: Glycosaminoglycan modification localizes CAIX to caveolin-1 in acidosis sensitive lipid raft domains. (A), Hypoxic U87-MG cells were incubated with M75 α-CAIX (red), and transferrin (Tfn; green), dextran (Dxn; green) or cholera cytotoxin subunit B (Ctx-B; green), respectively, and analyzed for co-localization. Merged images display strong co-localization of α-CAIX with Ctx-B (yellow signals). **(B)**, Immunofluorescence staining of human glioblastoma tumor sections shows partial co-localization of caveolin-1 (Cav-1; red) and CAIX (green) in merged images (yellow). WT-CAIX **(C)** and hypoxic parental **(D)** U87-MG cells were incubated at neutral (pH 7.4) or acidic (pH 6.5) conditions for 3 h, stained for CAIX (green) and caveolin-1 (red) and analyzed for co-localization. (A-D), Data shown are representative of at least three independent experiments. Hoechst nuclear stain (blue). All images were captured on an Airyscan super resolution imaging system integrated with Zeiss 710 confocal microscope. Scale bars, 10 µM (A, C, and D) and 50 µM (B). **(E)**, FACS quantification of α-CAIX internalization shows greater acidosis-mediated induction of α-CAIX uptake in WT-CAIX as compared with S54A-CAIX expressing cells. Figures above bars indicate fold change in acidosis as compared with neutral pH. Data shown are representative of two independent experiments, each performed in triplicates.
